# Low serum vitamin D levels increase the mortality of cardiovascular disease in older adults: A dose–response meta-analysis of prospective studies

**DOI:** 10.1097/MD.0000000000016733

**Published:** 2019-08-23

**Authors:** Jun Yang, Juan Ou-Yang, Ji Huang

**Affiliations:** a Department of Medical College, Tianmen Vocational College; b Department of Laboratory; c Department of Neurology, Tianmen First People's Hospital, Tianmen, Hubei Province, China.

**Keywords:** 25-hydroxyvitamin D, cardiovascular disease, dose–response relationship, meta-analysis

## Abstract

Lower circulating vitamin D is common in older adults and may be a potential reversible risk factor for cardiovascular disease (CVD) in older adults, however, presented controversial results.

Database was searched update to February 2018. Key data were extracted from eligible studies. Dose–response meta-analysis were conducted for synthesizing data from eligible studies.

A total of 13 eligible studies involving 21,079 participants were included in this meta-analysis. Person with lower 25-hydroxyvitamin D status (25 (OH)D level <50 nmol/L) appeared to have higher mortality of CVD in older adults (RR = 1.54, 95% CI 1.24–1.91). Furthermore, a significantly higher mortality of CVD in older adults was observed for the deficient (<25 nmol/L; RR = 1.47, 95% CI 1.15–1.81) and insufficient (25–50 nmol/L; RR = 1.16, 95% CI 1.04–1.27) categories of 25 (OH)D, compared to the reference category of >75 nmol/L. Additionally, decrease of 10 nmol/L 25-hydroxyvitamin D was associated with a 7% incremental in the risk of CVD mortality in older adults.

Considering these promising results, circulating vitamin D is associated with CVD mortality increment in older adults.

## 1 Introduction

Cardiovascular disease (CVD) is a major public health crisis in both developed and developing countries and is the leading cause of morbidity and mortality in the world.^[1–3]^ It is well established the role of traditional risk factors cannot completely explain the development of CVD, which has caused people to continue to look for new risk factors.^[4]^ In recent years, there has been increasing evidence that vitamin D deficiency is positively associated with the risk of CVD,^[5,6]^ as well as hypertension,^[7]^ obesity^[8]^ and cancer.^[9]^ Vitamin D deficiency is also shown to affect the progression of atherosclerosis.^[10]^

Vitamin D is a steroid hormone whose main function is to regulate the balance of calcium and phosphorus metabolism by acting on parathyroid glands, kidneys, and intestines. Although vitamin D can be ingested through food, it mainly comes from the synthesis of human body in vivo. Lower serum vitamin D is common in older adults^[11,12]^ and may be a potential reversible risk factor for CVD in older adults.^[13]^

The evidence on the relation between serum vitamin D and risk of CVD mortality in older adults is still inconclusive. Thus, we performed a dose–response meta-analysis to summarize and prospectively quantify the relative risk of low circulating 25-hydroxyvitamin D concentration and CVD mortality in older adults, and to provide an evidence-based reference for clinical use.

## 2 Methods

There are no ethical issues involved in our study for our data were based on published studies.

### 2.1 Search strategy

Cardiovascular diseases were defined as heart failure, myocardial infarction, ischemic heart disease, and stroke. Eligible studies were systematically searched of Medline, Embase, Web of Science, and Cochrane Database update to February 2018 with keywords including “Cardiovascular Disease” [MeSH] OR “Heart Failure” [MeSH] AND “Myocardial Infarction” [MeSH] OR “Ischemic Heart Disease” [MeSH] OR “Stroke” [MeSH] AND “Vitamin D” [MeSH] OR “25-hydroxyvitamin D” [MeSH] OR “25OHD” [MeSH] OR “Hypovitaminosis D” [MeSH] OR “1,25-dihydroxyvitamin D” [MeSH] AND “Older Adult” [MeSH] OR “Elderly” [MeSH].

### 2.2 Study selection

Then, the study was screened for retrieval based on the following criteria:

1. outcome must be CVD in older adults;
2. circulating 25-hydroxyvitamin D;
3. the relative risks with 95% confidence intervals;
4. age were ≥60.

### 2.3 Data extraction

The following study information was extracted from each eligible study: first author, publication year, study design, country, age, no of participants. Quality assessment was performed according to the Newcastle–Ottawa scale.^[14]^

### 2.4 Statistical analysis

Dose–response meta-analysis using the method recommended by Greenland, Longnecker, and Orsini and colleagues by using STATA software 14.0.^[15]^

## 3 Results

### 3.1 Literature search results

**Figure 1:**
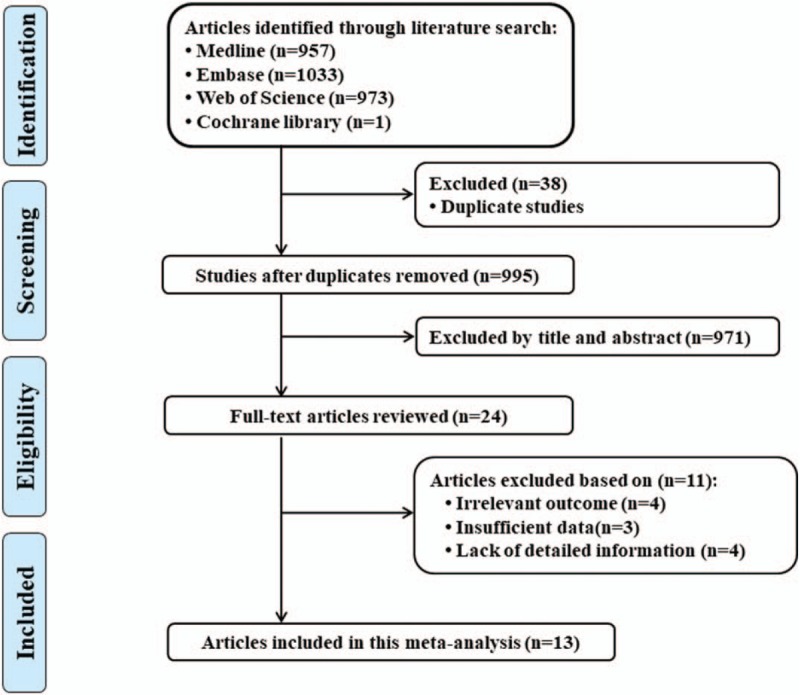
Flow diagram of the study selection process.

Figure 1 displayed the results of literature searching. A total of 957 studies were identified from Medline, 1033 studies were identified from Embase, 973 studies were identified from Web of Science, 1 study was identified from Cochrane Database. A total of 995 studies were identified after duplicates. Through screening of the title and abstract, 971 studies were excluded. Then, we download and read the remaining 24 studies. Among them, 4 studies were excluded due to no relevant outcome measure; 3 studies were excluded due to insufficient data; 4 studies were excluded due to lack of detailed information. Finally, 13 studies were used for the final data synthesis.^[16–28]^ The characteristics of the included studies are shown in the Tables 1 and 2.

**Table 1:**
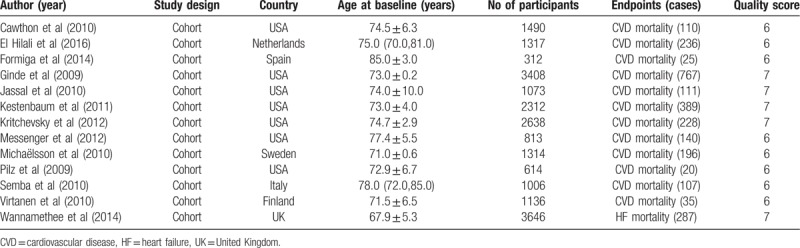
Characteristics of participants in included studies.

**Table 2:**
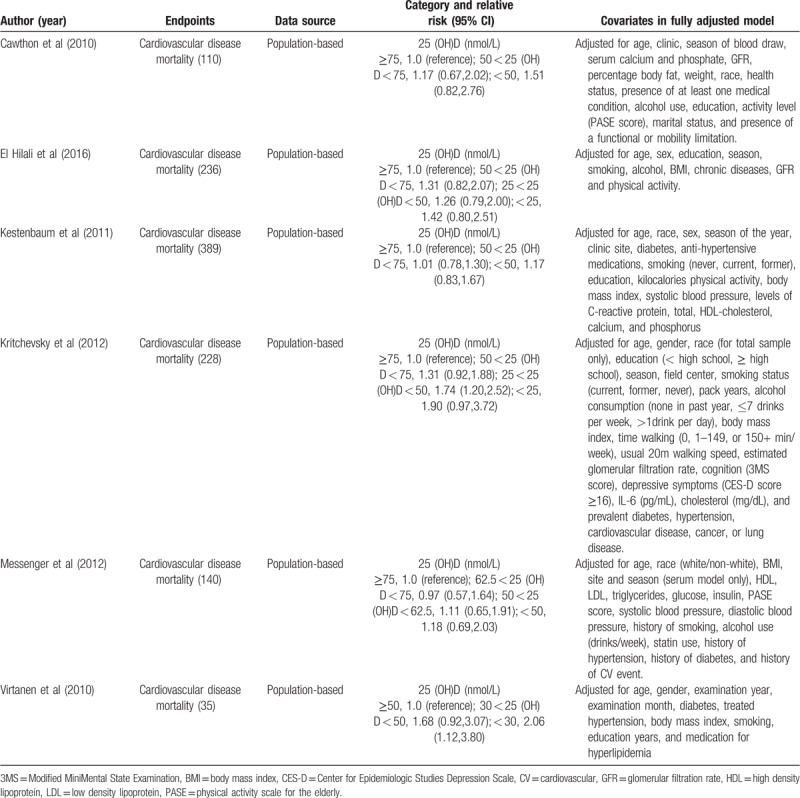
Outcomes and covariates of included studies.

### 3.2 Circulating vitamin D and risk of CVD mortality in older adults

**Figure 2:**
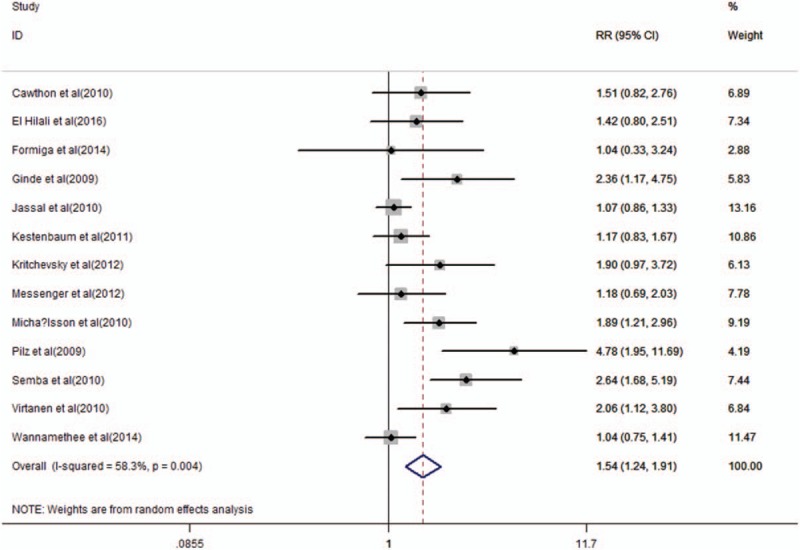
Forest plot showing the pooled effects of serum 25-hydroxyvitamin D on the risk of cardiovascular disease mortality in older adults. Solid diamonds and horizontal lines represent RRs (95% CIs) for the outcome of interest. Solid circles and horizontal lines represent RRs (95% CIs); the gray boxes reflect the statistical weight of the study. The dotted vertical line denotes the point estimate for the pooled RRs and the solid vertical line indicates the line of no effect. The open diamond represents the pooled RR with its 95% CI. CI = confidence interval; RRs = relevant risks.

Figure 2 displayed the results of circulating vitamin D and mortality of CVD in older adults. Person with lower 25-hydroxyvitamin D status (25 (OH)D level < 50 nmol/L) appeared to have higher risk in CVD mortality in older adults (RR = 1.54, 95% CI 1.24–1.91).

The definition of vitamin D deficiency is controversial. However, the majority believe that serum 25 (OH)D < 20 ng/mL (or 50 nmol/L) suggests that vitamin D is deficiency, serum 25 (OH)D between 20 and 30 ng/mL (or 50–75 nmol/L) suggests that vitamin D is insufficient, and serum 25 (OH)D between >30 ng/mL (or nmol/L) represents vitamin D is adequate.^[29]^ We found a significantly higher risk of CVD mortality in older adults was observed for the deficient (<25 nmol/L; RR = 1.47, 95% CI 1.15–1.81) and insufficient (25–50 nmol/L; RR = 1.16, 95% CI 1.04–1.27) categories of 25 (OH)D, compared to the reference category of >75 nmol/L (Table 3).

**Table 3:**
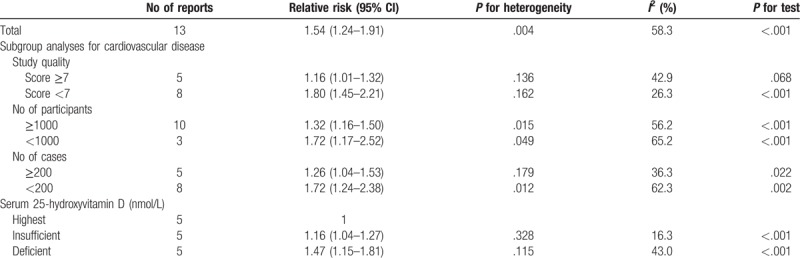
Stratified analyses of relative risk of cardiovascular disease mortality.

### 3.3 Dose–response meta-analyses between circulating vitamin D and risk of CVD mortality in older adults

**Figure 3:**
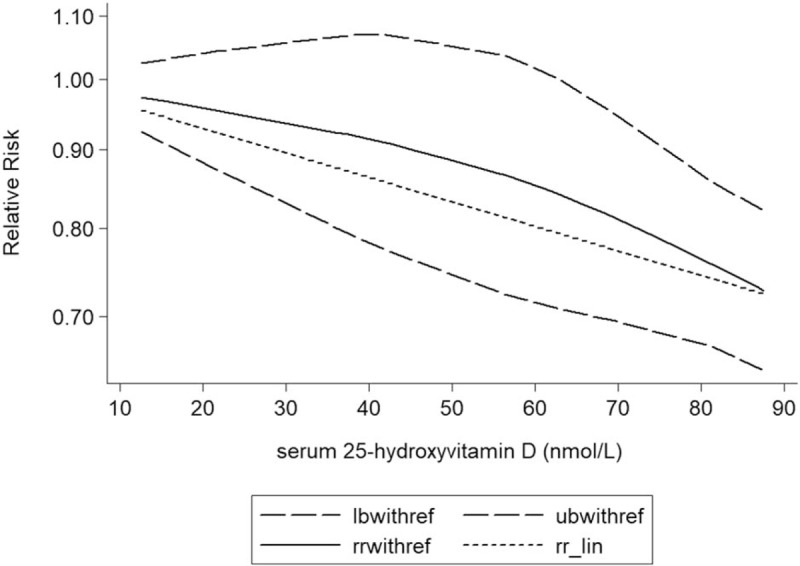
Dose–response analysis between serum 25-hydroxyvitamin D and the relative risk of cardiovascular disease mortality in older adults. The solid line represents point estimates of the association of serum 25-hydroxyvitamin D and cardiovascular disease risk with the use of a restricted cubic splines model, and the dashed lines indicate 95% CIs. CI = confidence interval; RRs = relevant risks.

The test for a nonlinear dose–response relationship was significant (likelihood ratio test, *P* < .001), suggesting curvature in the relationship, decrease of 10 nmol/L 25-hydroxyvitamin D was associated with a 7% incremental in the risk of CVD mortality in older adults, the summary relative risk of CVD mortality in older adults for an decrease of 10 nmol/L vitamin D was 1.07 (95%CI: 1.03, 1.12, *P* < .001) (Fig. 3).

### 3.4 Publication bias

**Figure 4:**
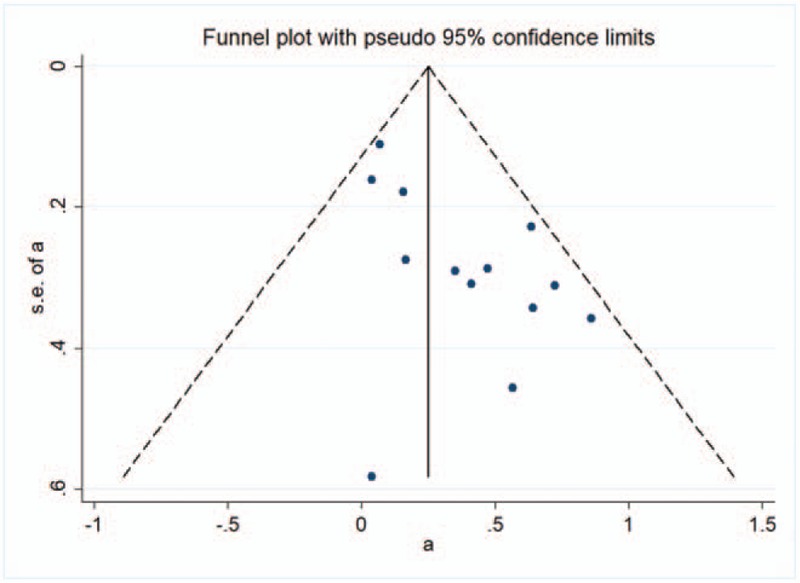
A funnel plot for the meta-analysis between serum 25-hydroxyvitamin D and the relative risk of cardiovascular disease mortality in older adults.

The result of circulating vitamin D and mortality of CVD in older adults funnel plots did not reveal any evidence of apparent asymmetry (Fig. 4). No significant publication bias was observed.

## 4 Discussion

Cardiovascular disease is a chronic disease caused by multiple risk factors. In the last 20 years, with the aging of the population, high blood pressure, the number of patients with diabetes is increasing, coupled with sedentary exercise, the morbidity and mortality rate of CVDs is rising obviously, which has seriously endangered the life and quality of life of the people. In addition to traditional risk factors, it has been noted that lower vitamin D levels may be a potential risk factor for CVD.^[6]^

Vitamin D is the essential nutrient of the human body. The body cannot be synthesized by itself, and the main source of vitamin D is UV synthesis.^[29]^ Vitamin D through interaction with vitamin D receptor (VDR), and VDR widely exists in the human body many kinds of cells, such as skeletal muscle cells, myocardial cells, pancreatic B cells, vascular endothelial cells, nerve cells, immune cells, and osteoblasts, suggesting that vitamin D is in addition to the classic regulation of calcium and phosphorus balance and maintain bone health, but also has other more bone wide biological effects.^[30]^ In recent years, the research on the maintenance of human health on the role of vitamin D increases, more and more evidence that vitamin D is not only a significant benefit for the prevention of osteoporosis and fractures, and may reduce the risk of cancer, infection, autoimmune diseases, cardiovascular system, and nervous system disease incidence rate.^[7,10]^ At present, vitamin D has neuroprotective effects, which can control the proinflammatory cytokines inducing cognitive impairment, participate in the synthesis of acetylcholine in neurotransmitters,^[31,32]^ and also relate to atherosclerosis, cerebral infarction, diabetes and hypertension.^[11]^

The complex mechanism of intracellular vitamin D has been established. 1,25-(OH)_2_D_3_ is the main form of biological activity in the human body of vitamin D_._ 1,25-(OH)_2_D_3_ in the blood as a free body or binding protein in the form of delivery to target cells, rapid integration with the cytoplasm of VDR, the corresponding DNA sequence is then transported to the nucleus and retinoic acid receptors X (RXR) formation of 1,25-(OH)_2_D_3_-VDR-RXR complex effect on the target gene, and the expression of structural gene regulation, which can be directly or indirect regulation of transcription of more than 200 genes, including the production of renal renin, the secretion of insulin in pancreas, the secretion of thyroid hormone, the production of macrophage cathelicidin factor.^[33]^ Since about 3% genome of human body is directly or indirectly regulated by vitamin D endocrine system, vitamin D deficiency can be directly or indirectly harmful to human health. With the aging of the population, the research of older adults has become a hot topic.^[34]^ Vitamin D deficiency is very common among the elderly, and the relationship between vitamin D and risk of CVD mortality in older adults is contradictory.

Previous meta-analysis based on 34 studies has found that lower circulating vitamin D level is associated with risk of CVD.^[35]^ However, there is no study to investigate the relationship between lower circulating vitamin D levels and risk of CVD mortality in older adults. Considering newly results identifying the relationship between serum vitamin D levels and risk of CVD mortality in older adults, we conducted a dose–response meta-analysis to summarize and prospectively quantify the relative risk of low circulating 25-hydroxyvitamin D concentration and CVD mortality in older adults. Thus, this meta-analysis provides the most up-to-date epidemiological evidence supporting lower circulating vitamin D is associated with CVD mortality increment in older adults.

Lower circulating vitamin D correlated with CVD mortality increment in older adults is biologically understandable. In the course of CVD, insulin resistance, diabetes, hypertension, abnormal lipid metabolism, obesity, and other risk factors play a very important role, and vitamin D levels play an important role in CVD risk factors. On the one hand, 1,25-(OH)_2_D_3_ regulates the expression of immune genes and apoptotic genes, which protects the immune damage of islet B cells, and at the same time reduces the apoptosis of islet B cells. On the other hand, it regulates insulin secretion and release by reducing calcium concentration in islet B cells. Vitamin D also regulates the expression of insulin receptor, the sensitivity of insulin to glucose transport and improving insulin sensitivity.^[36]^ During follow-up, it was found that the incidence of diabetes decreased by 40% in the highest baseline vitamin D group compared with those in the baseline vitamin D lowest group.^[37]^ Kayaniyil et al prove that vitamin D can improve insulin resistance and increase insulin secretion.^[38]^ Secondly, the level of 1,25-(OH)_2_D_3_ was negatively correlated with blood pressure.^[39]^ On the one hand, 1,25-(OH)_2_D_3_ can regulate the expression of vascular endothelial growth factor through vitamin D response element, upregulate the activity of vascular endothelial nitric oxide synthase, increase the synthesis of prostacyclin, and promote vasodilation. On the other hand, the vasoconstriction of vascular endothelium can be inhibited by preventing calcium ions from flowing into vascular endothelial cells. Therefore, when vitamin D is deficient or deficient in the body, the vasodilation function of vascular endothelium decreases, leading to a rise in blood pressure.^[40]^ Thirdly, inflammation plays an important role in the occurrence of CVD, and reducing the occurrence of inflammatory reaction plays an important role in preventing CVD.^[41]^ It has been found that vitamin D has anti-inflammatory and immunomodulatory functions, especially that 1,25-(OH)_2_D_3_ can down-regulate the expression of various inflammatory factors in immune cells, such as interleukin-1, interleukin-6, interleukin-8, and α-tumor necrosis factor.^[42]^ Nuclear factor NF-κB is an important modulator in the process of inflammatory factor synthesis. 1,25-(OH)_2_D_3_ can down-regulate NF-κB by binding to VDR.^[43]^ In addition, vitamin D can also induce the expression of Iκ Ba protein.^[44]^ Fourth, Vitamin D is related not only to insulin secretion and insulin resistance, but also to low density lipoprotein cholesterol (LDL-C) and total cholesterol.^[45,46]^ Carbone et al found that serum 1,25-(OH)_2_D_3_ level in the experimental group was positively correlated with lipoprotein A-I levels, and negatively correlated with the LDL-C/HDL-C ratio.^[47]^ There are still some other mechanisms that need further study.

Our study has a number of limitations. Because most studies do not report the use of quality assessment schemes, the relationship between circulating vitamin D concentration and CVD mortality in older adults may be confounded by assay variations across studies. Second, study duration was short in these studies and person included in these studies may be different from the real life. Third, the overall sample size of the study was small, which may have a certain impact on the evaluation results.

Our findings underscore the notion that circulating vitamin D is associated with CVD mortality increment in older adults. Correcting vitamin D deficiency may prevent the occurrence of CVD mortality. This will be a simple, economical, and safe method. Can supplement vitamin D be used as a preventive or therapeutic drug for CVD? In the future, large-scale and ongoing trials must be performed in the future to validate the risk identified in the current meta-analysis.

## Acknowledgments

This work was received no funding

## Author contributions

**Conceptualization:** Ji Huang.

**Data curation:** Jun Yang, Juan Ou-Yang, Ji Huang.

**Formal analysis:** Jun Yang, Juan Ou-Yang, Ji Huang.

**Methodology:** Jun Yang.

**Writing – original draft:** Ji Huang.

**Writing – review & editing:** Ji Huang.
